# Didactics of written argumentation with Spanish as a Foreign Language (SFL) students at university level in Algeria

**DOI:** 10.3389/fpsyg.2023.1192823

**Published:** 2023-04-21

**Authors:** Saida Baaziz, María Isabel de Vicente-Yagüe Jara

**Affiliations:** ^1^Department of German, Spanish and Italian, University of Argel 2, Algiers, Algeria; ^2^Department of Didactics of Language and Literature, University of Murcia, Murcia, Spain

**Keywords:** argumentation, essay, Spanish as a Foreign Language (SFL), microstructure, macrostructure, superstructure

## Abstract

The general objective of this research is to investigate the impact of a program focusing on the development of argumentation techniques on the improvement of critical essays by SFL students at the University of Algiers 2. A quasi-experimental pretest-posttest study was carried out, where the indicators of textual levels obtained by the students were evaluated and compared, both before and after a learning intervention. The study involved 126 students studying SFL at the University of Algiers 2, whose essays were analyzed using Parodi and Núñez’s (1999) analysis guidelines, in order to evaluate the students’ argumentative competence at the three levels of microstructure, macrostructure and superstructure. Once the results were analyzed, statistically significant differences were observed in the indicators of the macrostructure, and superstructure. Furthermore, an improvement in the post-test means is observed in the indicators of these microstructure. Lastly, the intervention program strengthened students’ argumentative abilities, which had a significant effect on their critical essays.

## 1. Introduction

Education is the fundamental foundation for a country’s progress; its goal is to develop students’ skills so that they can be responsible citizens who actively engage in social life. Considering the continuous transformation of today’s society, it is necessary to incorporate knowledge and skills in the university curriculum in order to provide students with the opportunity to develop their communication skills, which will enable them to function in the productive world and provide them with creativity and dynamism. In this context, Pipkin and Reynoso (2010) note that teaching argumentation is currently one of the fundamental topics of pedagogical reflection. However, traditional pedagogical models that focus on the accumulation of information and the repetition of formulas become very passive practices in the classroom and do not allow for interaction between subjects, which results in didactic tasks becoming meaningless. For this reason, the didactic development of argumentation must be recognized as a fundamental skill to replace the passive transmission of knowledge with an interactive discourse between teacher and student (Obando, 2007). Therefore, it is necessary to overcome the shortcomings of traditional pedagogical models, abandoning their behaviorist and decontextualized patterns in favor of an integrative model that pursues reflection and argumentative interaction in strategic and contextualized situations. This methodological innovation in the didactics of argumentation is committed to learner autonomy and creativity, replacing the formal rigidity that has dominated the pedagogical scenario in traditional textual commentary.

In the same vein, Gómez Barriga (2012) argues that argumentation is a practice that can be found in different communicative contexts: at work, in everyday and academic conversations. In the university context, it constitutes a tool for the dissemination and generation of knowledge that contributes to the human and social development of the learner. Thus, Gómez Barriga (2012) asserts that the ability to produce arguments in all cultures is a key factor for success in politics, work, community, and family. There is an urgent need for the appropriate use of argumentation to express opinions in a climate of tolerance, respect, and consideration for the position of others, in order to reach agreements without reaching violent confrontations (Díaz and Mujica, 2007). For his part, Serrano (2008) emphasizes that the ability to argue enables students to act with good judgment in solving problems by presenting well-founded and convincing arguments.

Similarly, Ruiz et al. (2015) state that argumentation is a fundamental tool for the learning process, as it helps students to meaningfully understand the concepts addressed, in addition to promoting an interactive environment of academic debate and discussion. Specifically, Ruiz et al. (2015) propose a model of teaching argumentation that includes three types of relationships: epistemic (developing argumentation for knowledge construction), conceptual (the skill requires the use of dialogue, debate, criticism, decision making, listening, and respect), and *didactic* (language allows for the exchange of meanings and concerns).

According to the above, the argumentative text should be a frequently used resource throughout the teaching and learning process of students, and especially in the development of the subject of Spanish as a Foreign Language (SFL). Various studies have already focused on the study of the argumentative essay, providing theories and procedures for improving its practice in the classroom (Parodi, 2000; Díaz, 2002; Padilla et al., 2011; Bañales et al., 2015; Caro et al., 2018; Castro and Sánchez, 2018; Vicente-Yagüe et al., 2019, 2023; Baaziz, 2022; Caro and Vicente-Yagüe, 2022).

It should be noted that the production of argumentative texts is a cognitive process related to the operations of critical thinking, because it aims to identify a problem, understand, propose solutions, compare, contrast, analyze, evaluate, synthesize, establish causal relationships, and point out consequences. Díaz (2002) emphasizes that learning to write is at the same time learning to think critically and creatively, given that we develop our ability to think critically when we learn to write academic texts.

If we analyze the different educational contexts regarding the didactics of argumentation, the study by Camps (1995) in the Spanish context examines the different obstacles that hinder students in writing argumentative texts. First, he mentions the difficulty of understanding a text, taking into account aspects such as the author’s intention, the recipient and the reader’s own social situation. In addition, there is the difficulty of identifying their own opinions on different topics, understanding the counterargument, using concessions to defend an opposing thesis, and insufficient knowledge of linguistic resources.

In the Venezuelan context, the research by Sánchez and Álvarez (1999) on the development of written argumentative skills stands out; their results highlight the lack of argumentative structure in students’ texts, as well as the absence of strategies for expressing their own opinions. Serrano and Villalobos (2008), for their part, verify the absence of discursive procedures in the production of written texts. Serrano’s (2001) study points out inconsistencies at the superstructure level of the written texts produced by the students.

In the Chilean context, the study by Núñez (1999) shows results of low performance in structural development after analyzing the elaborated writing essays. Similarly, Parodi and Núñez (1999) research on the evaluation of argumentative writing production stands out, where the students’ difficulties in elaborating their texts are discussed in relation to the three levels of textual competence.

In the Argentine context, Perelman (2001) addresses students’ writing difficulties and mentions their problems with textual structuring, lack of knowledge of argumentative strategies to support opinions, and insufficient resources to connect the text. In the Mexican context, the work of Castro and Sánchez (2018) stands out; they report the following writing problems: ignorance of the function and value of textual quotations to support their arguments, imitation of the position of other voices without developing their own voice (giving their personal appreciation as an extension of the expert opinion to validate their own discourse), ignorance of the discursive resources that allow the author’s positioning.

In the Algerian context, the study by Bellatrèche (2013), among the studies on the didactics of French at the University of Mostaganam, highlights the unsatisfactory argumentative competence of students in the three textual dimensions. Moreover, the results obtained by Belaouf (2016) show that students have difficulties at the linguistic level, at the level of coherence, and at the level of argumentative competence; the study confirms that the difficulties appear early in school and persist until university education.

The present research is aimed at the Algerian university context in the learning and teaching of Spanish as a Foreign Language (SFL). Considering that argumentation is a textual typology that should be worked on by students throughout their academic career, it is appropriate to carry out research in the two Spanish degree programs at the University of Algiers 2.

Therefore, the general objective of the present research is to study the effect of the intervention of a program focused on the development of argumentation strategies on the improvement of the development of critical essays by SFL students at the University of Algiers 2. This general objective is articulated by the following specific objectives:

– To compare the grades obtained by the students in the indicators of the microstructure level, before and after the didactic intervention.
– To compare the grades obtained by the students in the indicators of the macro-structural level, before and after the didactic intervention.
– To compare the grades obtained by the students in the indicators of the superstructure level, before and after the didactic intervention.

The proposed hypothesis is that the students will obtain better results in the written essays after carrying out a didactic intervention in their class sessions on the process of argumentative writing.

## 2. Research method

### 2.1. Design

A pre-experimental pretest-posttest study (Campbell and Stanley, 2005) was conducted to evaluate and compare indicators of students’ writing levels before and after a learning intervention focused on discursive argumentation strategies for critical essay writing.

### 2.2. Participants

The study included 126 students enrolled in the second and third degrees of Spanish as a Foreign Language at the University of Algiers 2, between the ages of 19 and 22, as shown in Table 1. All students in both courses were included in the study without any special selection. A non-probabilistic purposive sampling procedure was used, based on convenience (Sáez, 2017).

**Table 1:** Participants in the study.

Level	Gender	Number of students	Total
2nd	Masculine	5	71
	Feminine	66	
3rd	Masculine	7	55
	Feminine	48	
Total			126

### 2.3. Research instrument

The analysis guide by Parodi and Núñez (1999, pp. 74-77) was used to evaluate students’ argumentative competence in the texts produced (Table 2). The instrument follows the classification of Van Dijk (1992), which explains three textual levels: microstructure, macrostructure, and superstructure. Microstructure is a set of interrelated and coherent propositions that make up the different sentences of a text. Macrostructure refers to a set of propositions that synthesize the overall meaning of the text and are necessary for textual coherence. The superstructure represents the way in which the information of the text is organized according to a scheme whose components are the thesis, a series of arguments, and a conclusion.

**Table 2:** Written production analysis guideline.

Level	Indicators and criteria	
Microstructure	Thematic progression	
	Adequate maintenance of thematic progression, without breaks	3
	Maintenance of thematic progression with one break	2
	Maintenance of thematic progression with more than one break	1
	Inter-sentential relations	
	Coherent relations between sentences	3
	Inter-sentential relations with one break	2
	Inter-sentential relations with more than one break	1
Macrostructure	Topic	
	Adequate maintenance of the assigned theme	3
	Maintenance of assigned topic with one break	2
	Maintenance of assigned topic with more than one break/developed topic not relevant to the task	1
	Macro propositions with argument function	
	2 or more macro propositions with argument function consistent with each other	3
	2 or more macro propositions with argument function in the form of a list/only one macro proposition with argument function	2
	2 or more macro propositions, inconsistent with each other/no macro proposition with argument function	1
Superstructure	Thesis	
	Thesis included in the text and relevant to the task.	3
	Semi-explicit thesis	2
	Absence of thesis or thesis not relevant to the task	1
	Argumentation	
	An argument justified by supporting facts and consistent with each other	3
	An insufficiently justified argument	2
	Absence of argument/an unsupported argument	1
	Conclusion	
	Inclusion of argumentative conclusion	3
	Argumentative conclusion semi-explained or partially derived from the above	2
	Absence of argumentative conclusion/conclusion not pertinent to thesis or arguments	1

The indicators that correspond to each of the levels are articulated in three criteria that are scored on a scale of 1 to 3. The instrument allows us to evaluate the capacities achieved by the students and to measure their performance at each textual level, classifying their productions in the performance criteria established in the guideline. In short, it assesses the students’ ability to organize and produce coherent and cohesive argumentative texts.

### 2.4. Procedure

The procedure of the present research is articulated in the didactic intervention program and in the two moments before and after the moment of written production of an essay by the students. Thus, first, the students in the 2nd and 3rd years must write an essay on “The incorporation of ICT in university education” and “The university facing the online challenge of the coronavirus,” respectively, which will be analyzed as a pretest; then, the program is applied in the classroom, whose sessions conclude with the writing of a new essay, which constitutes the posttest.

The didactic intervention program is presented in Table 3 and was validated by five expert judges who validated its content (degree of precision and conceptual, syntactic, and structural adequacy) with respect to the purpose of the research. The evaluations offered were discussed and agreed upon, with suggestions for improvement for its final version.

**Table 3:** Didactic intervention program.

Phases	Sessions	2nd course	3rd course
Initial	1	Theoretical session	Theoretical session
	2	Argumentative production begins on the obligatory nature of vaccination against Covid 19	Initial argumentative production on euthanasia
Workshop	3	Analytical reading of text (Covid-19 denial) and collaborative revision of writings.	Analytical reading of text (Euthanasia: Covid-19 vaccine) and collaborative revision of writings.
	4	Thesis statements and argumentative debates	Thesis statements and argumentative debate
	5	Textual cohesion activities	Textual cohesion activities
	6	Text analytical reading: Influencers: a double reality for greenwashing	Text analytical reading: gender-based violence
	7	Text analytical reading: The Capital Punishment	Text analytical reading: The Capital Punishment
	8	Collaborative writing and textual revision	Collaborative writing and textual revision
Final	9	Argumentative production in a control situation	Argumentative production in a control situation
	10	Comprehension and written production in a test. Topic: The fakes news about Covid-19	Comprehension and written production in a test. Topic: Islamophobia

The program is divided into three phases: introductory phase, argumentative workshop, and final phase. Throughout the program, the cognitive, structural, and linguistic dimensions of written composition are addressed, including sessions with different aspects: text comprehension, planning, individual argumentative writing, revision and rewriting for self-evaluation and self-criticism, oral debates, and collaborative argumentative writing in groups for peer learning and fostering a cooperative environment. In addition, the didactic intervention carried out with the students was developed in a total of 20 sessions, throughout the academic year. Specifically, 10 sessions were dedicated to each of the courses, distributed in two sessions per week, during 5 months of the first semester of the course.

The study complied with the ethical guidelines of the University of Algiers 2 required for research on human subjects: informed consent, right to information, protection of personal data, guarantee of confidentiality, non-discrimination, free of charge, and the possibility of withdrawing from the program at any stage.

### 2.5. Data analysis

First, the corpus of texts written by the students was analyzed according to the criteria and indicators of the textual levels presented by Parodi and Núñez (1999, pp. 74-77).

Second, once the scores were calculated, the Kolmogorov–Smirnov normality test was applied to determine the relevance of using parametric tests. This test showed that the different indicators of textual levels (*p* < 0.05) were not distributed according to the normal distribution. Therefore, the Wilcoxon test was used to compare the scores obtained by the students before and after the didactic intervention.

## 3. Results

The results are presented according to the specific objectives, each of them related to three textual levels: microstructure, macrostructure, and superstructure. To this end, Table 4 shows the scores obtained for each of the indicators of the textual levels analyzed.

**Table 4:** Pre-test and post-test results for students in the 2nd and 3rd courses.

LEVEL	Indicators	Pre test				Post test			
		2nd course		3rd course		2nd course		3rd course	
		*M*	DT	*M*	DT	*M*	DT	*M*	DT
Microstructure	Thematic progression	1.02	0.62	1.2	0.65	1.3	0.52	1.4	0.66
	Inter-sentential relationships	1.1	0.81	1.2	0.45	1.6	0.34	1.7	0.47
Macrostructure	Topic	1.1	0.72	1.3	0.64	1.9	0.65	2.2*	0.56
	Macro-propositions with argument function	1.2	0.54	1.2	0.44	2.2**	0.85	2.4**	0.86
Superstructure	Thesis	1.1	0.67	1.2	0.61	2.8***	0.67	2.6***	0.56
	Argumentation	1.2	0.86	1.4	0.75	2.2**	0.86	2.4**	0.58
	Conclusion	1.1	0.53	1.2	0.32	2*	0.57	2.3*	0.46

*p < 0.05, **p < 0.01, ***p < 0.001.

With respect to the first specific objective, which was to compare the students’ scores on the microstructural level indicators before and after the didactic intervention, no statistically significant differences were observed in the thematic progression and inter-sentence relations in either of the two grades of the study sample. However, there was an improvement in the posttest means of the 2nd and 3rd grades for both indicators.

Therefore, in terms of microstructure, the students do not manage to overcome their initial difficulties, they remain in a low performance of the criteria. It is not possible to find a significant positive effect after the intervention, as it is verified the persistence of errors in their writing. The writings are incoherent and diffuse, due to several errors of nominal and verbal co-reference, particularly the lack of concordance between the number of the nominal element and the pronouns that substitute it, the lack of gender concordance, the lack of concordance between the number of the nominal element and the corresponding verb tenses or the use of incongruent verb tenses. Regarding the component of inter-sentence relations, there are numerous errors in the use of linguistic and semantic mechanisms.

Regarding the second specific objective, which focused on comparing the scores of the macro-structural indicators, statistically significant differences are found in the topic indicator for students in grade 3, although the improvement of students in grade 2 can also be observed. There are also statistically significant differences in the macro-propositions with argument function indicator for both classes of students.

The data obtained from the macrostructure in the pretest show that most of the students have difficulties in thematic development due to the insufficient use of mechanisms for hierarchizing information and relating ideas, which hinders text comprehension. Regarding the students’ use of macro-sentences, there are no arguments, but their use is limited to definitions or explanations of the topics of the given instructions. However, the post-test data show evolutionary differences, and an achievement is observed at all levels of performance, as the given topics are adequately developed with coherent arguments.

Finally, it is verified that in both courses there are statistically significant differences at all levels of the superstructure (thesis, argumentation, and conclusion). In the post-test, after the development of the didactic intervention, there are notable evolutionary differences, because an adequate development of the topics of the given instructions is demonstrated, in such a way that explicit theses are presented, supported with clear arguments, and converge in the enunciation of adequate conclusions.

As for the dimension of the superstructure in the pretest, the data showed that the students had enormous difficulties in formulating its components. Regarding the thesis, most of them were unable to state their position for or against the topic. Similarly, the writings showed deficiencies in the formulation of arguments and reasons to support the thesis. Likewise, the conclusion showed a lack of knowledge and poor command of how to present the final conclusion of the argumentative writing. On the contrary, the analysis of the texts produced after the didactic intervention shows a significant achievement in all the indicators of the superstructure.

## 4. Conclusion

After analyzing the results, it can be observed that after the intervention program focused on the development of argumentative writing strategies, a significant effect was obtained in the students’ critical essays. The argumentative skills of the study sample were strengthened after the didactic intervention, as improvements and a positive effect were observed in the three textual levels. Although there was a slight improvement in the microstructure, it is the level with the most inconsistent indicators in the framework of writing competence. However, it is necessary to note that, given the lack of a control group in the design of this research, there may have been other variables that influenced the observed improvements, in addition to the intervention program itself. This issue should be pointed out as a limitation of the study and leads the research on the didactics of argumentation towards other types of methodological designs that allow us to confirm the results obtained with the present study sample.

Written argumentation is considered a difficult task that involves various thought processes, from gathering the necessary information, to defending positions with valid evidence, to formulating pertinent conclusions. Therefore, it is of paramount importance for the teacher to support the learner through an active teaching process based on persuasive tasks so that the student achieves argumentative competence, in addition to insisting on the primordial value of reading in argumentative writing (Vicente-Yagüe et al., 2023). Thus, this study highlights the valuable recovery of the argumentative practice in the classroom and the pedagogical reconversion aimed at new approaches to writing based on critical reflection, overcoming academic models focused on the meaning of the text and the author’s intention.

It is worth noting that, in recent years, several research studies carried out in different countries have shown the importance of promoting the mastery and use of argumentation skills in university students, both orally and in writing. Their contributions constitute theories for the development of learners’ skills and the improvement of their ability to persuade, argue and acquire a more complex language so that they are able to elaborate persuasive speeches for their interlocutors. It should be noted that several researchers have already pointed out that the successful completion of a written composition is not an easy task and requires a specific intervention that allows students to master the grammatical, linguistic and discursive processes of argumentation (Álvarez, 1995, 2001; Camps, 1995; Perelman, 2001; Díaz, 2002; Martínez, 2002; Obando, 2007; Matteucci, 2008; Serrano, 2008; Ramírez, 2010; Fernández Millán et al., 2021).

For this reason, the present study focuses on written argumentation, which is addressed in the university curriculum of the SFL degree at the University of Algiers 2. In fact, this skill is essential for the development of students’ critical thinking and the promotion of skills that allow them to express their points of view on a given topic, to communicate and express their ideas and opinions in writing according to their own criteria, and to defend them with valid arguments (Parodi, 2000). In fact, its use is of paramount importance, since it allows them to access information, process it and take a stand in different situations that arise in their learning process (Vicente-Yagüe et al., 2019).

In this sense, it is appropriate to emphasize that the lack of studies on written composition in general and argumentative typology in the Algerian university classroom in particular justifies the need to develop studies in the classrooms themselves with a university sample through designs that include didactic interventions. A rethinking of university studies in SFL is necessary in order to incorporate efficient didactic proposals in all academic courses that address the mismatches in the three textual levels and allow addressing students’ difficulties in the direction of developing their written argumentative competence. This type of research is necessary to promote students’ argumentative discourse in the broad field of written production.

## Data availability statement

The raw data supporting the conclusions of this article will be made available by the authors, without undue reservation.

## Author contributions

The research structure and the theoretical framework were designed by the SB and MV-Y. SB designed the didactic intervention and collected the results, under the supervision of MV-Y. MV-Y focused on the methodology, writing, and the translation into English. All authors contributed to the article and approved the submitted version.

## Conflict of interest

The authors declare that the research was conducted in the absence of any commercial or financial relationships that could be construed as a potential conflict of interest.

## Publisher’s note

All claims expressed in this article are solely those of the authors and do not necessarily represent those of their affiliated organizations, or those of the publisher, the editors and the reviewers. Any product that may be evaluated in this article, or claim that may be made by its manufacturer, is not guaranteed or endorsed by the publisher.
